# Time-Dependent Diagnostic Windows After Isolated Diffuse Axonal Brain Injury in Rats

**DOI:** 10.3390/diagnostics16162503

**Published:** 2026-08-08

**Authors:** Vladislav Zvenigorodsky, Benjamin F. Gruenbaum, Ilan Shelef, Dmitry Frank, Beatris Tsafarov, Shahar Negev, Shuly Doviner, Amit Frenkel, Michael Dubilet, Alexander Zlotnik, Matthew Boyko

**Affiliations:** 1 Department of Radiology, Soroka University Medical Center and the Faculty of Health Sciences, Ben-Gurion University of the Negev, Beer-Sheva 84101, Israel; zvenigorodsky@gmail.com (V.Z.); shelef@bgu.ac.il (I.S.); 2 Department of Anesthesiology and Perioperative Medicine, Mayo Clinic, Jacksonville, FL 32224, USA; gruenbaum.benjamin@mayo.edu; 3 Department of Anesthesiology and Critical Care, Soroka University Medical Center, Ben-Gurion University of the Negev, Beer-Sheva 84101, Israel; frdima16@gmail.com (D.F.); shahar.shoshani@gmail.com (S.N.); frenkela@clalit.org.il (A.F.); michaeldu@clalit.org.il (M.D.); zlotnika@bgu.ac.il (A.Z.); 4 Department of Histology, Soroka University Medical Center and the Faculty of Health Sciences, Ben-Gurion University of the Negev, Beer-Sheva 84101, Israel; beatriceza@clalit.org.il; 5 Faculty of Health Sciences, Ben-Gurion University of the Negev, Beer-Sheva 84101, Israel; shulydov@post.bgu.ac.il

**Keywords:** β-amyloid precursor protein, diffuse axonal brain injury, diffusion tensor imaging, fractional anisotropy, hippocampus, traumatic brain injury

## Abstract

**Background:** Diffuse axonal brain injury (DABI) is a major component of traumatic brain injury, but the optimal timing of magnetic resonance imaging and histologic assessment after isolated DABI remains unclear. Because each method captures a different phase of injury, defining time-dependent diagnostic windows may improve experimental design and reduce animal use. **Methods:** Sixty-four Sprague-Dawley rats were randomized to moderate DABI or sham operation. Neurological Severity Score (NSS), 3 T diffusion tensor imaging (DTI), and histologic assessment were performed at early and late time points. Hippocampal fractional anisotropy (FA) was measured at 48 h and 1 month. β-amyloid precursor protein (β-APP) immunohistochemistry assessed acute axonal injury at 48 h, and hematoxylin and eosin (H&E) staining quantified hippocampal neuronal density at 1 month. **Results:** At 48 h, DABI rats had higher NSS, lower hippocampal FA, and greater β-APP-positive axonal accumulation than sham-operated rats. By 1 month, NSS and hippocampal FA no longer differed between groups, whereas hippocampal neuronal density remained significantly reduced in DABI rats, indicating persistent structural injury. **Conclusions:** In this rat model of isolated DABI, hippocampal FA and β-APP immunohistochemistry identified acute axonal pathology at 48 h, whereas conventional histology detected a persistent reduction in neuronal density at 1 month. These findings support a time-optimized diagnostic strategy using early DTI for noninvasive injury detection and late histology for long-term structural assessment.

## 1. Introduction

Traumatic brain injury (TBI) is a major global health problem and remains a leading cause of death and long-term disability worldwide [1,2]. TBI accounts for up to one-third of accidental deaths, and many survivors develop persistent neurological, cognitive, and psychiatric sequelae that substantially impair quality of life [1,2]. Diffuse axonal brain injury (DABI), caused by rapid rotational or translational forces that shear long axonal fibers, is one of the most common pathological patterns of TBI and is estimated to occur in approximately 40–50% of cases [3,4]. Clinically, DABI is associated with coma, prolonged cognitive and behavioral impairment, and poor functional outcome [3,5].

DABI is difficult to isolate in patients because it often occurs alongside other TBI-related pathology, including edema, hemorrhage, contusions, and blood–brain barrier disruption. This makes it challenging to define the specific contribution of axonal injury to long-term neurological and psychiatric outcomes [5]. To address this limitation, rotational acceleration models in rats have been developed to produce isolated DABI without macroscopic cortical injury. These models allow more precise study of axonal pathology, behavioral consequences, and potential therapeutic strategies [4,5,6].

Neuropathological assessment remains the reference standard for confirming DABI. Immunohistochemical markers of disturbed axonal transport, particularly β-amyloid precursor protein (β-APP) and related amyloid markers, can detect injured axons within hours to days after rotational injury. Prior work from our group and others has shown that β-APP or amyloid-β-related immunohistochemistry sensitively identifies axonal injury in selected brain regions, including the hippocampus [4]. However, these methods are terminal and require perfusion, tissue processing, and separate animal cohorts for each time point. Conventional histology is less sensitive to early axonal transport failure but is useful for assessing later structural consequences, including neuronal loss, gliosis, and tissue remodeling. It also requires terminal sampling [3,4].

Magnetic resonance imaging (MRI) provides a complementary, noninvasive approach. Diffusion tensor imaging (DTI) offers quantitative measures of tissue microstructure that are particularly relevant to DABI [4,6]. Fractional anisotropy (FA), which reflects the directional coherence of water diffusion, decreases in regions of axonal disruption and has emerged as a sensitive MRI marker of DABI-related injury [4,6]. Using a 3-T clinical scanner and our rotational DABI model, we previously established a practical MRI protocol and showed that FA reductions in deep gray structures, including the hippocampus, are robust markers of injury that can match or exceed histological sensitivity at early time points [4,6]. We have also shown that isolated DABI produces persistent depressive- and anxiety-like behaviors in a substantial subset of animals, supporting the functional relevance of this pathology [5].

Despite these advances, the time-dependent diagnostic performance of MRI-based FA, early amyloid-related immunohistochemistry, and later conventional histology remains incompletely defined after isolated DABI. In particular, the temporal relationship between acute FA changes, early amyloid-related axonal pathology, and later structural alterations has not been systematically characterized within a single experimental framework. This gap complicates the design of long-term mechanistic and therapeutic studies, where investigators must select methods and time points that balance sensitivity, feasibility, animal use, and the need for longitudinal assessment.

The present study was designed to define time-dependent diagnostic windows after isolated DABI in rats. Using our established rotational injury model, 3-T MRI protocol, and histological methods, we examined hippocampal FA as a noninvasive imaging marker, early amyloid-related immunostaining as a sensitive acute marker of axonal injury, and later conventional histology as a marker of persistent structural damage during the first month after injury [4,5,6]. Our objective was to determine how the sensitivity and applicability of these complementary approaches change over time and to define a time-optimized combination of MRI and histological techniques for assessing hippocampal pathology after isolated DABI.

## 2. Materials and Methods

### 2.1. Animals

The study included 64 Sprague-Dawley rats, consisting of 32 males and 32 females, obtained from Harlan Laboratories, Israel. Animals weighed 280–320 g at the time of the experiment. Rats were housed under standard laboratory conditions with a 12 h light/dark cycle, controlled temperature of 22 ± 1 °C, and free access to Purina Chow and water. All experimental procedures were performed during the dark phase between 08:00 and 16:00. All experiments were approved by the Animal Care Committee of Ben-Gurion University of the Negev, Israel, and were conducted in accordance with institutional and international guidelines for the care and use of experimental animals (BGU-317-03-2024C and IL-11-02-2019C).

### 2.2. Experimental Design

**Figure 1:**
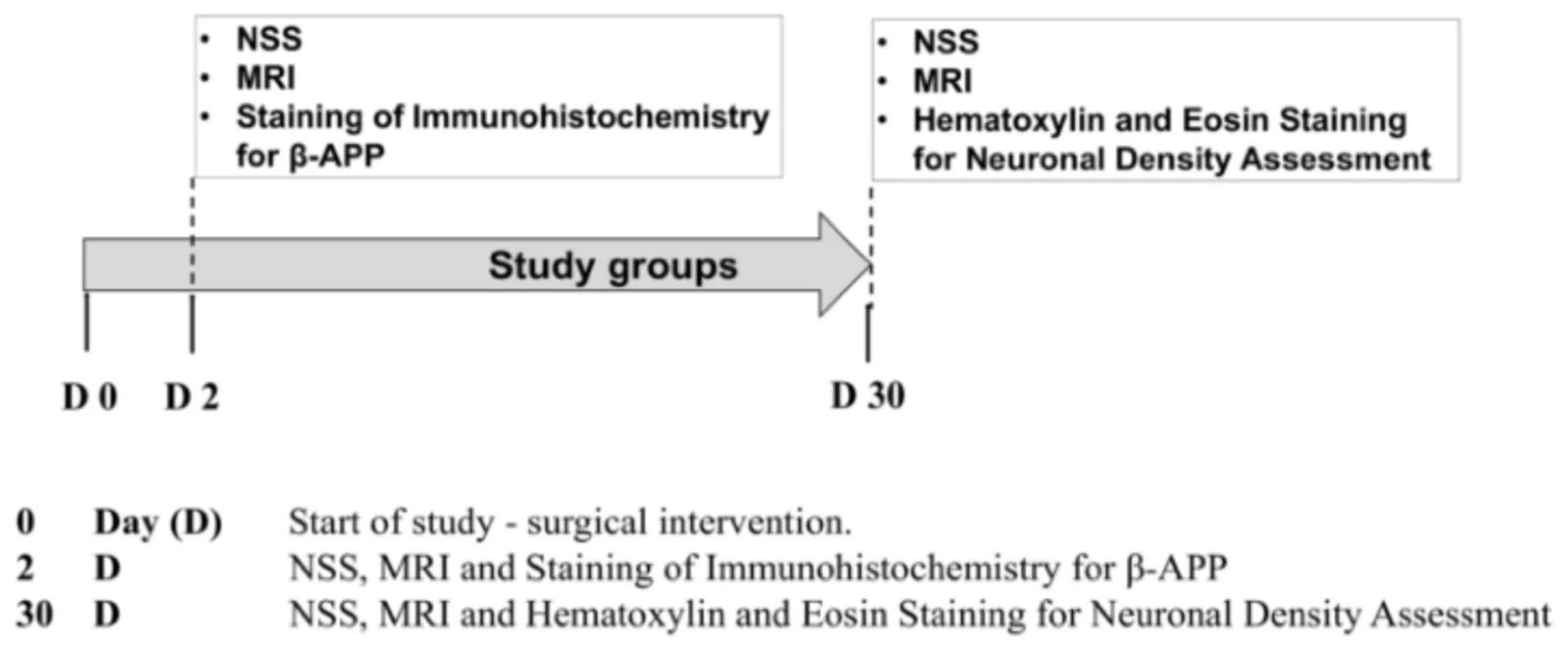
Experimental timeline. All animals underwent NSS and MRI at 48 h. After the 48 h MRI, the early cohort underwent terminal β-APP immunohistochemistry. The remaining animals underwent NSS and MRI at 1 month followed by terminal H&E assessment of hippocampal neuronal density. β-APP and H&E were performed in separate cohorts.

The study used a 2 × 2 factorial design with treatment group (sham or DABI) and sex (male or female) as factors. A total of 64 rats were randomly assigned to the sham-operated group (*n* = 32) or the DABI group (*n* = 32), with 16 males and 16 females in each group. Within each treatment and sex group, eight animals were randomly allocated to the 48 h terminal cohort and eight were randomly allocated to the 1 month terminal cohort. All 64 rats underwent Neurological Severity Score (NSS) assessment at baseline and NSS and MRI at 48 h. After the 48 h MRI, the early cohort (*n* = 32) was perfused for β-APP immunohistochemistry. The remaining 32 rats underwent NSS and MRI at 1 month and were then perfused for hematoxylin and eosin (H&E) assessment of hippocampal neuronal density. Thus, β-APP and H&E were performed in separate terminal cohorts. Animal allocation is summarized in Table 1, and the experimental timeline is shown in Figure 1.

### 2.3. Neurological Severity Score

Neurological function was assessed using the NSS, as previously described [5,7,8]. Evaluations were performed by two independent observers blinded to group allocation. Scores were assigned based on motor and behavioral performance, with 0 indicating normal neurological function and 15 indicating maximal neurological impairment. The NSS included assessment of gait on a wide surface using a 3-point scale, gait on a narrow surface using a 4-point scale, effort to remain on a narrow surface using a 2-point scale, beam walking using a 3-point scale, and beam balance using a 3-point scale.

### 2.4. Induction of Moderate DABI

Moderate DABI was induced using a rotational acceleration device previously developed and described by our group [4,8]. The apparatus consists of four main components: a transparent cylindrical chamber, a weighted iron mass, a rotational assembly containing a cylindrical tube, dual bearings, and a head fixation unit with ear pins, and a horizontal platform supported by two bearings. Rats were anesthetized with isoflurane, 5% for induction and 1.5–2.5% for maintenance, delivered in a 1:1 mixture of medical air and oxygen. The animal’s head was secured using ear pins placed in the external auditory canal on the side of impact. A 2 kg weight was released from a height of 150 cm. Impact of the falling weight triggered the rotational mechanism, causing rapid rotation of the head from 0° to 90°. After the procedure, animals were monitored in a recovery chamber until fully awake.

### 2.5. β-APP Immunohistochemistry

The β-APP immunohistochemistry was performed to quantify β-APP-positive axons as a marker of acute axonal injury. Tissue was collected at the early post-injury time point after MRI acquisition. Under deep anesthesia, the thoracic cavity was opened and animals were perfused through the left ventricle with cooled saline at a pressure of approximately 110 mmHg until clear fluid was obtained from the right atrium. This was followed by perfusion with 250 mL of 4% paraformaldehyde in 0.1 M phosphate-buffered saline, pH 7.4. Brains were removed immediately and fixed in 4% buffered paraformaldehyde for 48 h at 4 °C. The brains were divided into 5 mm coronal slices from the olfactory bulb to the visual cortex. After paraffin embedding, 5-μm sections were cut using a microtome. Immunohistochemical staining and analysis were performed as previously described [6,7].

### 2.6. H&E Staining for Neuronal Density

Conventional H&E histology was performed 1 month after DABI or sham intervention, immediately after MRI acquisition [9]. Under deep anesthesia, animals underwent transcardial perfusion with cooled saline at approximately 110 mmHg, followed by 250 mL of 4% paraformaldehyde in 0.1 M phosphate-buffered saline, pH 7.4. Brains were removed and post-fixed in 4% paraformaldehyde in 0.1 M phosphate-buffered saline (pH 7.4) for at least 48 h. After fixation, tissue was dehydrated through graded alcohols, cleared in xylene, and embedded in paraffin. Coronal sections of 5 μm thickness containing the hippocampal formation were mounted on glass slides and stained with H&E according to routine laboratory protocols. Slides were digitized using a whole-slide scanner and archived in the Sectra IDS7 PACS application, Sectra AB, Linköping, Sweden. Neuronal density in the hippocampal cornu ammonis 1 (CA1) region was quantified under the supervision of an experienced neurohistopathologist who was blinded to group allocation. For each animal, six non-overlapping square regions of interest (ROIs) were placed within the CA1 pyramidal layer on a representative section. Each ROI measured 0.39 mm × 0.39 mm, based on the spatial calibration of the IDS7 viewer. Neurons were counted manually on high-magnification digital images. Only cells with visible nucleoli and preserved neuronal morphology were included. The number of neurons per field was divided by the field area and adjusted for section thickness, 5 μm, to calculate neuronal density in neurons/mm^3^. The same ROI placement strategy, inclusion criteria, and counting rules were applied across all groups. Tissue processing and slide preparation were performed by an experienced technician from the Pathophysiology Laboratory, Soroka University Medical Center, Be’er Sheva, Israel.

### 2.7. MRI Acquisition

Diffusion tensor MRI was performed at 48 h and 1 month after DABI or sham intervention using a 3-T clinical MRI scanner (Ingenia, Philips Medical Systems, Best, The Netherlands) with an eight-channel receive-only coil, as previously described [4,7]. A clinical 3-T scanner was used to enhance the translational relevance of the protocol and to determine whether reliable imaging markers of DABI could be obtained using equipment commonly available in clinical settings [4]. Acquisition parameters, including voxel size, were optimized to maintain adequate signal-to-noise ratio and allow reproducible delineation of deep gray structures in the rat brain while preserving a scan duration feasible for a clinical 3-T protocol [10].

Animals were maintained under general anesthesia with 1.5% isoflurane in oxygen during image acquisition. Diffusion tensor imaging was performed in the axial plane using a multi-shot spin-echo echo-planar imaging sequence. Imaging parameters were as follows: repetition time/echo time = 1419/138 ms, echo-planar imaging (EPI) factor = 19, sensitivity encoding (SENSE) factor = 1.5, b value = 1000 s/mm^2^, six diffusion directions, and spectrally selective fat suppression. Seven slices were acquired with no interslice gap. Spatial resolution was 0.55 × 0.55 × 2.0 mm, and five signal averages were obtained, resulting in a total scan time of 11 min 19 s.

Post-processing was performed using the IntelliSpace Portal workstation (version 5.0.0.20030, Philips Medical Systems, Best, The Netherlands). FA maps were generated using the Philips MRI software platform (version 5.7.1.2; Ingenia, Philips Medical Systems, Best, The Netherlands). For each predefined region of interest, diffusion tensor imaging metrics were calculated and averaged for statistical analysis.

### 2.8. Regions of Interest and DTI Parameter Map Analysis

For group-level analysis, images were transformed into a common anatomical space by constructing a nonlinear composite brain from individual scans. ROIs were manually delineated on the average brain using the Paxinos and Watson Rat Brain Atlas as an anatomical reference [11]. Bilateral hippocampal ROIs were defined in the CA1 stratum pyramidale. DTI metrics were calculated from the slice at bregma −3.14 mm and averaged across the left and right hippocampal ROIs for each animal.

FA maps were generated using the Philips MRI software platform, version 5.7.1.2, Ingenia, Philips Medical Systems, Best, The Netherlands. Image analysis was performed by two experienced evaluators blinded to group allocation. For each predefined ROI, DTI metrics were calculated and averaged for statistical analysis [4,12,13].

To assess reliability, inter-rater agreement was evaluated in a subset of 18 scans and showed excellent agreement for hippocampal measurements, intraclass correlation coefficient (ICC) > 0.88. All MRI datasets underwent quality control before analysis. Scans with predefined quality concerns, including excessive motion artifacts or signal dropout, were excluded.

### 2.9. Statistical Analysis

Statistical analyses were performed using IBM SPSS Statistics for Windows, version 31.0.1.0 (IBM Corp., Armonk, NY, USA). Continuous variables were assessed for normality using the Kolmogorov–Smirnov test. Homogeneity of variance was evaluated using Levene’s test. Data from the 2 × 2 factorial design were analyzed using two-way analysis of variance (ANOVA), with treatment group (sham-operated or DABI) and sex (male or female) as fixed factors. The primary outcomes included MRI parameters, hippocampal neuronal density, and the number of β-APP-positive axons. When assumptions for parametric testing were met and a significant main effect or interaction was identified, group differences were further evaluated using estimated marginal means with Bonferroni-adjusted pairwise comparisons. NSS was first evaluated using two-way ANOVA to assess the effects of treatment group, sex, and their interaction. Because NSS is an ordinal outcome, between-group comparisons after pooling males and females were performed using two-sided Mann–Whitney U tests at each time point. Parametric data are presented as mean ± standard deviation (SD), and nonparametric data are presented as median and interquartile range (IQR). Cohen’s d was calculated using the pooled standard deviation. A *p* value < 0.05 was considered statistically significant in two-tailed tests.

## 3. Results

### 3.1. Neurological Severity Score

At 48 h after injury, two-way ANOVA showed a significant main effect of group on NSS, with higher scores in DABI rats than in sham-operated rats (Table 2). Sex and the sex × group interaction were not significant. The overall model was significant, F(3,60) = 18.666, *p* < 0.001, partial η^2^ = 0.483. Levene’s test indicated unequal variances across groups, F = 14.534, *p* < 0.001; however, the group effect remained robust. Because no sex-related differences were detected, male and female animals were pooled for the final comparison. Median NSS was 0 (0–0.25) in sham-operated rats and 2.5 (1.0–3.25) in DABI rats. The between-group difference was significant by Mann–Whitney U test, U = 108, *p* < 0.001, r = 0.714. At 1 month, two-way ANOVA showed no significant effect of group, sex, or sex × group interaction on NSS. The overall model was not significant, F(3,28) = 0.163, *p* = 0.920. After pooling male and female animals, NSS remained low and comparable between sham-operated and DABI rats, median 0 (0–0.0) versus 0 (0–0.25), U = 120, *p* = 0.693, r = 0.070.

### 3.2. Hippocampal Neuronal Density

**Figure 2:**
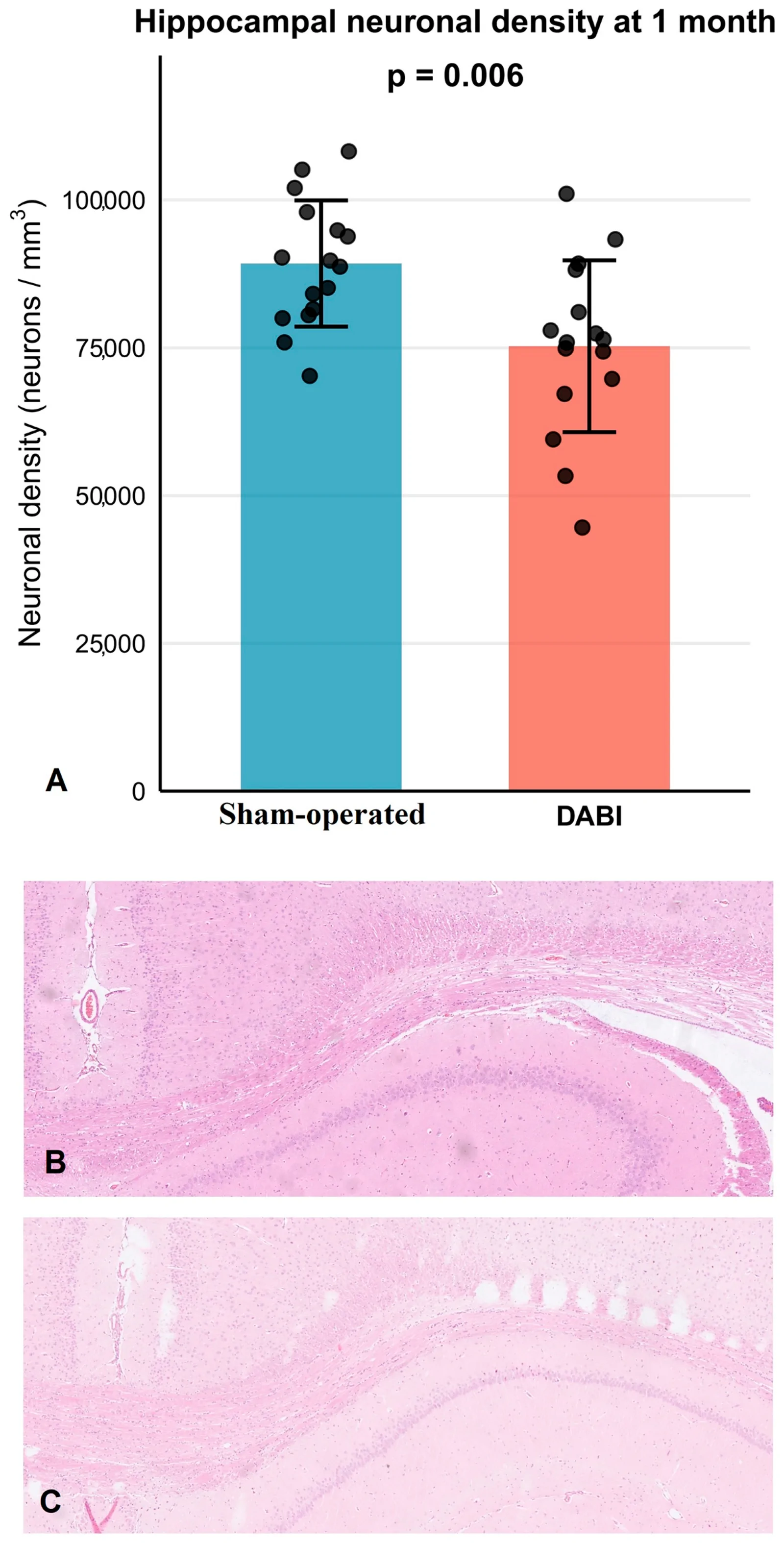
(**A**) Hippocampal neuronal density at 1 month. Representative Panel (**C**) shows individual CA1 neuronal density values. Bars represent means, error bars represent standard deviations, and each point represents one animal, *n* = 16 per group. Neuronal density was lower in DABI rats than in sham-operated rats, *p* = 0.006. H&E-stained sections from a sham-operated rat (**B**) and a DABI rat (**C**) are shown. Panels (**B**,**C**) are shown at ×50 magnification.

At 1 month after injury, two-way ANOVA showed a significant main effect of group on hippocampal neuronal density in H&E-stained sections. Sex and the sex × group interaction were not significant. The overall model was significant, F(3,28) = 3.023, *p* = 0.046, partial η^2^ = 0.245. Levene’s test did not indicate heterogeneity of variance, F = 0.309, *p* = 0.819. Because no sex effect was detected, male and female animals were pooled. Hippocampal neuronal density was significantly lower in DABI rats than in sham-operated rats, 75,256.41 ± 14,527 versus 89,262.82 ± 10,674 neurons/mm^3^, F(1,28) = 9.028, *p* = 0.006, partial η^2^ = 0.244 (Figure 2).

### 3.3. β-APP Accumulation in the Hippocampus

**Figure 3:**
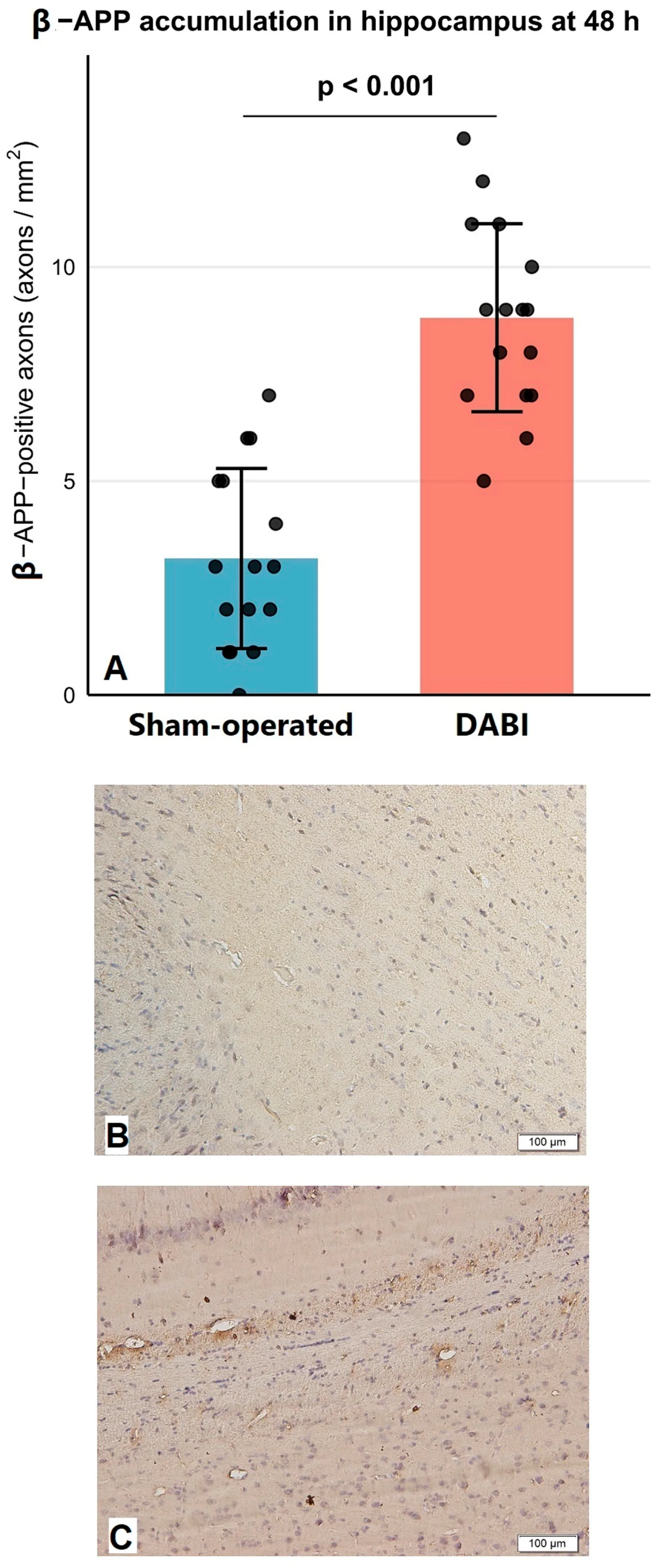
β-APP-positive axons in the hippocampus at 48 h. Panel (**A**) shows individual β-APP-positive axon density values. Bars represent means, error bars represent standard deviations, and each point represents one animal, *n* = 16 per group. DABI rats had greater β-APP-positive axon density than sham-operated rats, *p* < 0.001. Representative β-APP immunohistochemical sections from a sham-operated rat (**B**) and a DABI rat (**C**) are shown. Scale bar in panel (**B**) and (**C**) = 100 μm.

At 48 h after injury, two-way ANOVA showed a significant main effect of group on the number of β-APP-positive axons in the hippocampus. Sex and the group × sex interaction were not significant. The overall model was significant, F(3,28) = 17.131, *p* < 0.001, partial η^2^ = 0.647. Levene’s test supported homogeneity of variance across groups, F(3,28) = 0.222, *p* = 0.880. Because neither sex, F(1,28) = 0.101, *p* = 0.753, partial η^2^ = 0.004, nor the group × sex interaction, F(1,28) = 0.025, *p* = 0.875, partial η^2^ = 0.001, was significant, male and female animals were pooled. The number of β-APP-positive axons was higher in DABI rats than in sham-operated rats, 8.81 ± 2.20 versus 3.19 ± 2.10 axons/mm^2^. The adjusted between-group difference was significant, mean difference = 5.63, standard error (SE) = 0.79, *p* < 0.001, Bonferroni-adjusted (Figure 3).

### 3.4. Hippocampal Fractional Anisotropy

At 48 h after injury, two-way ANOVA showed a significant main effect of group on hippocampal FA. Sex and the sex × group interaction were not significant. The overall model was significant, F(3,60) = 20.857, *p* < 0.001, partial η^2^ = 0.510. Levene’s test indicated homogeneity of variance, F(3,60) = 1.578, *p* = 0.204. Mean FA was significantly lower in DABI rats than in sham-operated rats, 0.273 ± 0.0457 versus 0.379 ± 0.0610. The adjusted between-group difference was significant, mean difference = 0.106, SE = 0.014, *p* < 0.001, Bonferroni-adjusted. The group effect was present in both males, 0.276 ± 0.014 versus 0.368 ± 0.014, *p* < 0.001, and females, 0.269 ± 0.014 versus 0.390 ± 0.014, *p* < 0.001. No sex difference was detected within either group.

**Figure 4:**
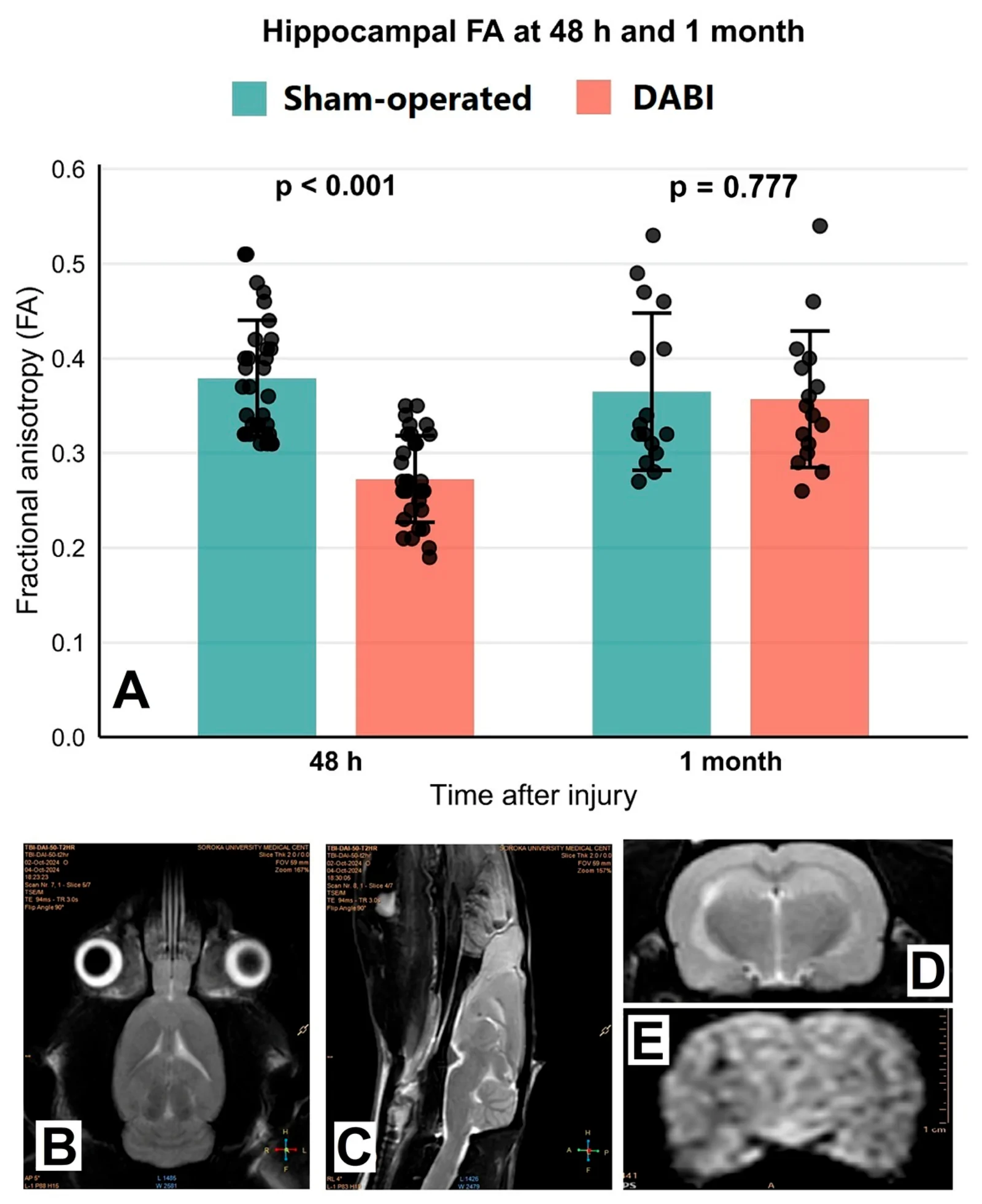
Hippocampal fractional anisotropy at 48 h and 1 month. Panel (**A**) shows individual bilateral hippocampal FA values at 48 h and 1 month. Bars represent means, error bars represent standard deviations, and each point represents one animal, *n* = 32 per group at 48 h and *n* = 16 per group at 1 month. FA was lower in DABI rats than in sham-operated rats at 48 h (*p* < 0.001) but did not differ between groups at 1 month (*p* = 0.777). Representative axial (**B**), sagittal (**C**), and coronal (**D**) magnetic resonance images and a representative coronal diffusion tensor image (**E**) are shown.

At 1 month, hippocampal FA no longer differed between groups. Two-way ANOVA showed no significant effect of group, sex, or sex × group interaction. The overall model was not significant, F(3,28) = 0.029, *p* = 0.993, partial η^2^ = 0.003. Mean FA was similar in sham-operated and DABI rats, 0.365 ± 0.082 versus 0.357 ± 0.072, mean difference = 0.008, SE = 0.028, *p* = 0.777, Bonferroni-adjusted. FA also did not differ between males and females, 0.362 ± 0.081 versus 0.360 ± 0.075, *p* = 0.948 (Figure 4).

### 3.5. Comparative Effect Sizes of the Primary Outcomes

Standardized effect sizes were calculated to compare the magnitude of the differences between the sham-operated and DABI groups across the three primary outcomes. β-APP-positive axon counts at 48 h showed the largest effect size (Cohen’s d = 2.61), followed by hippocampal FA at 48 h (Cohen’s d = 2.00) and hippocampal neuronal density at 1 month (Cohen’s d = 1.10). These values are presented as descriptive comparisons within this dataset and are not intended to define minimum group sizes for future studies.

## 4. Discussion

The main finding of this study is that MRI and histologic markers identify different diagnostic windows after isolated DABI. At 48 h, DABI produced a clear increase in NSS, a marked reduction in hippocampal FA, and increased β-APP-positive axons. By 1 month, NSS and hippocampal FA no longer differed between groups, but hippocampal neuronal density remained significantly reduced. These findings suggest that early FA and β-APP capture acute axonal injury and microstructural disruption, whereas H&E-based neuronal density reflects later structural consequences that remained detectable when the between-group difference in hippocampal FA was no longer present at 1 month.

At the early time point, the results were highly consistent across functional, imaging, and histologic measures. DABI rats had higher NSS than sham-operated rats, indicating measurable neurologic impairment 48 h after injury. At the same time, hippocampal FA was substantially lower in DABI rats, supporting the sensitivity of DTI to acute microstructural injury in this model. β-APP staining showed a parallel increase in injured axons, consistent with impaired axonal transport after rotational injury. The convergence of these findings is important because it suggests that FA measured on a clinical 3 T scanner is sensitive to early microstructural abnormalities occurring alongside β-APP-defined axonal transport disturbance [6,7,14].

The 1 month findings showed a different pattern. NSS and hippocampal FA no longer differed between DABI and sham-operated animals. In contrast, hippocampal neuronal density remained significantly lower in DABI rats. This dissociation suggests that the absence of a between-group difference in hippocampal FA does not indicate full tissue recovery. Instead, it may reflect resolution of the acute diffusion abnormalities associated with axonal injury while persistent structural injury remains detectable by conventional histology [13,15]. In the present study, FA distinguished DABI from sham animals at 48 h but not at 1 month within the selected CA1 ROI. This result should not be interpreted as evidence that FA is limited to the acute phase of DABI [16]. Similar dissociations have been reported in experimental TBI, where advanced diffusion measures, histology, or structural MRI provided information beyond conventional FA at selected time points [17,18,19].

These results help define the practical role of each modality. FA showed a strong early effect in this dataset and has the major advantage of being noninvasive. It can be repeated in the same animal, making it useful for longitudinal studies and for reducing the number of animals needed across time points. β-APP immunohistochemistry remains a valuable acute marker because it directly identifies axonal transport disturbance, but it is terminal and limited to early tissue sampling [20]. H&E-based neuronal density provides a measure of persistent structural injury at later time points but does not directly assess acute axonal transport failure. Because β-APP and H&E were evaluated in separate terminal cohorts and reflect different pathological features, the present study does not establish that the reduced neuronal density at 1 month represents a direct continuation of the β-APP-positive axonal pathology observed at 48 h.

The effect-size comparison supports this interpretation. β-APP-positive axon counts at 48 h showed the largest standardized group difference, followed by hippocampal FA at 48 h and hippocampal neuronal density at 1 month. The large effect size for β-APP-positive axon counts was expected because sham tissue showed limited axonal β-APP accumulation, whereas DABI produced marked accumulation consistent with impaired axonal transport. In this model, β-APP therefore supports the presence of acute axonal injury but does not, by itself, identify the etiology of TBI. These differences do not imply that one method is universally superior. Instead, each method addresses a different experimental question. Early FA and β-APP are markers of acute injury, whereas late neuronal density provides an endpoint for persistent tissue damage.

Taken together, the data support a time-optimized approach to experimental DABI assessment. An early DTI examination around 48 h can provide a sensitive, noninvasive measure of injury severity and may reduce the need for β-APP staining in every experimental arm. β-APP immunohistochemistry can then be reserved for selected early cohorts when mechanistic confirmation of axonal injury is needed. A later H&E or neuronal nuclei (NeuN)-based assessment at 1 month can serve as the structural endpoint, particularly when the goal is to determine whether an intervention prevents persistent neuronal loss or tissue remodeling. This strategy is consistent with prior MRI-histology and multi-outcome approaches in TBI and stroke models [6,7,10,21,22] and may reduce terminal sampling while preserving sensitivity across the acute and chronic phases of injury.

Several limitations should be considered. First, the study focused on the hippocampus, particularly the CA1 region. This choice was based on the vulnerability of CA1 to mechanically induced injury, its role in cognitive processing, and its relevance to long-term behavioral outcomes after TBI [23,24,25,26]. This focus was also informed by our prior DABI work showing FA reductions and β-APP accumulation across multiple deep and cortical structures, including the hippocampus, thalamus, neocortex, and corpus callosum [4,6,7]. However, DABI affects multiple gray and white matter regions, and the timing of imaging and histologic changes may differ across vulnerable structures, including the corpus callosum, thalamus, cortex, and brainstem [27,28]. Independent longitudinal studies have similarly demonstrated region-dependent evolution of diffusion and pathological abnormalities across the corpus callosum, thalamus, cortex, hippocampus, and other white-matter tracts [14,19]. The diagnostic windows identified here should therefore be considered specific to hippocampal CA1. Future studies should determine whether similar temporal patterns are present in other vulnerable regions.

Second, MRI was performed on a clinical 3 T scanner using a six-direction DTI protocol. This improves translational relevance because it reflects equipment and acquisition strategies that are more widely available than high-field experimental MRI, and is consistent with prior work showing strong MRI-histology correlations in experimental brain injury models [6,7,21,29]. However, the spatial and angular resolution of this protocol may limit sensitivity to subtle, localized, or asymmetric abnormalities, particularly because FA was averaged across bilateral hippocampal ROIs. It also cannot fully characterize complex fiber architecture. More advanced diffusion approaches, including higher spatial or angular resolution and multi-shell acquisitions, may detect additional abnormalities not captured here [17,30].

Third, the study used terminal tissue assessment at selected time points. As a result, individual trajectories linking early FA or β-APP changes to later neuronal density could not be directly assessed in the same animal. In addition, the late histological assessment was limited to H&E staining. Inclusion of complementary markers such as NeuN for neuronal integrity and glial fibrillary acidic protein (GFAP) or ionized calcium-binding adapter molecule 1 (Iba1) for glial responses could have provided additional insight into the cellular mechanisms underlying the persistent structural changes observed after DABI [18,31]. Although no significant sex effects were detected and pooling was statistically justified, the study may not have been powered to detect more subtle sex-specific differences in injury evolution. Finally, some analyses showed variance heterogeneity or involved multiple comparisons. The main group effects were large and biologically consistent, but confirmation in larger cohorts would strengthen these findings.

Despite these limitations, the study has practical implications for preclinical DABI research. Most clinical TBI includes both focal and diffuse injury, making it difficult to isolate the contribution of axonal pathology to long-term neurologic and psychiatric outcomes. This model allows that component to be studied more directly. Prior work in this same rotational DABI model showed persistent depressive and anxiety-like behaviors after isolated DABI [5]. The present study identified early FA and β-APP abnormalities and reduced hippocampal neuronal density at 1 month, even when hippocampal FA and NSS no longer differed between groups. These findings provide a framework for matching the diagnostic method to the biological phase of injury. For therapeutic studies, this may help investigators select endpoints more rationally: FA for early screening and longitudinal monitoring, β-APP for acute mechanistic validation, and H&E or NeuN-based neuronal density for long-term structural outcome.

Overall, this model showed different findings at the acute and chronic time points. Under the present protocol, hippocampal FA and β-APP immunohistochemistry distinguished DABI from sham animals at 48 h, whereas conventional histology identified reduced hippocampal neuronal density at 1 month. A combined strategy using early MRI and late histology may therefore provide a practical and efficient approach for studying the evolution of DABI and for testing interventions aimed at reducing long-term structural injury.

## 5. Conclusions

In this rat model of isolated DABI, early MRI and histologic measures identified distinct phases of injury. At 48 h, reduced hippocampal FA and increased β-APP-positive axons provided sensitive markers of acute axonal pathology. By 1 month, hippocampal FA and NSS no longer differed between groups, but hippocampal neuronal density remained significantly reduced. This finding indicates persistent structural injury that was not reflected by hippocampal FA under the present imaging protocol. These findings support a time-optimized diagnostic strategy in which early DTI is used for noninvasive detection of acute injury, β-APP immunohistochemistry is reserved for selected early mechanistic cohorts, and late H&E or NeuN-based histology is used to assess long-term structural outcome. This combined approach may improve experimental design, reduce unnecessary terminal sampling, and provide broader diagnostic coverage across the first month after DABI.

## Figures and Tables

**Table 1:** Animal allocation and assessments by treatment group, sex, and terminal time point. Animals assigned to the 48 h terminal cohort underwent β-APP immunohistochemistry after the 48 h MRI. Animals assigned to the 1 month terminal cohort underwent assessments at both 48 h and 1 month and were then processed for H&E histology. β-APP and H&E were performed in separate animals.

Treatment Group	Sex	Initial *n*	48 h NSS and MRI, *n*	β-APP at 48 h, *n*	1 Month NSS and MRI, *n*	H&E at 1 Month, *n*
Sham-operated	Male	16	16	8	8	8
Sham-operated	Female	16	16	8	8	8
DABI	Male	16	16	8	8	8
DABI	Female	16	16	8	8	8
Total		64	64	32	32	32

**Table 2:** **Neurological severity score at 48 h and 1 month after injury.** Data are presented as median and interquartile range. At 48 h, NSS was significantly higher in DABI rats than in sham-operated rats. At 1 month, no significant between-group difference was observed.

NSS Values of the Study Groups		
Animal Groups	48 h *n*, Median (IQR)	1 Month *n*, Median (IQR)
Sham-operated	*n* = 32, 0 (0–0.25)	*n* = 16, 0 (0–0)
DABI	*n* = 32, 2.5 (1–3.25)	*n* = 16, 0 (0–0.25)

## Data Availability

The raw data supporting the conclusions of this article will be made available by the authors on request.

## Abbreviations

The following abbreviations are used in this manuscript:

ANOVA	Analysis of variance
β-APP	β-amyloid precursor protein
CA1	Cornu ammonis 1
DABI	Diffuse axonal brain injury
DTI	Diffusion tensor imaging
DWI	Diffusion weighted imaging
EPI	Echo planar imaging
FA	Fractional anisotropy
GFAP	glial fibrillary acidic protein
H&E	Hematoxylin and eosin
Iba1	Ionized calcium-binding adapter molecule 1
ICC	Intraclass correlation coefficient
IQR	interquartile range
MRI	Magnetic resonance imaging
NeuN	neuronal nuclei
NSS	Neurological severity score
PACS	Picture archiving and communication system
PBS	Phosphate-buffered saline
ROI	Region of interest
SD	Standard deviation
SE	Standard error
SENSE	Sensitivity encoding
TBI	Traumatic brain injury
TE	Echo time
TR	Repetition time
